# Study of Cytotoxic Effects of Benzonitrile Pesticides

**DOI:** 10.1155/2015/381264

**Published:** 2015-08-03

**Authors:** Petra Lovecka, Marketa Thimova, Petra Grznarova, Jan Lipov, Zdenek Knejzlik, Hana Stiborova, Tjokorda Gde Tirta Nindhia, Katerina Demnerova, Tomas Ruml

**Affiliations:** ^1^Department of Biochemistry and Microbiology, University of Chemistry and Technology Prague, Technicka 3, 166 28 Prague 6, Czech Republic; ^2^Department of Mechanical Engineering, Engineering Faculty, Udayana University, Jimbaran, Bali 80361, Indonesia

## Abstract

The benzonitrile herbicides bromoxynil, chloroxynil, dichlobenil, and ioxynil have been used actively worldwide to control weeds in agriculture since 1970s. Even though dichlobenil is prohibited in EU since 2008, studies addressing the fate of benzonitrile herbicides in the environment show that some metabolites of these herbicides are very persistent. We tested the cytotoxic effects of benzonitrile herbicides and their microbial metabolites using two human cell lines, Hep G2 and HEK293T, representing liver and kidneys as potential target organs in humans. The cell viability and proliferation were determined by MTT test and RTCA DP Analyzer system, respectively. The latter allows real-time monitoring of the effect of added substances. As the cytotoxic compounds could compromise cell membrane integrity, the lactate dehydrogenase test was performed as well. We observed high toxic effects of bromoxynil, chloroxynil, and ioxynil on both tested cell lines. In contrast, we determined only low inhibition of cell growth in presence of dichlobenil and microbial metabolites originating from the tested herbicides.

## 1. Introduction

**Figure 1:**
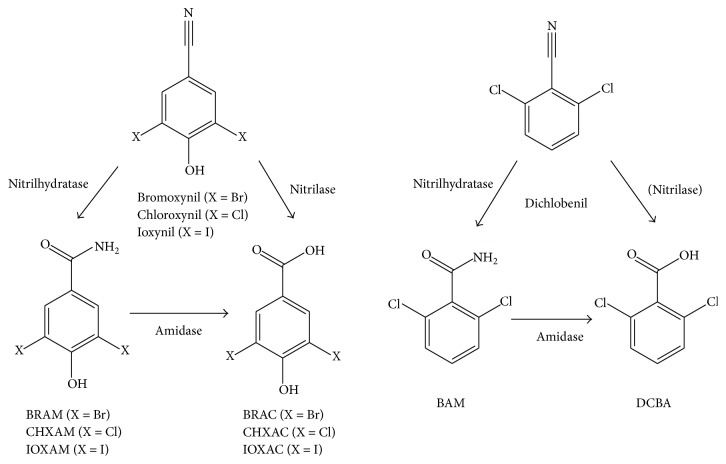
Hydrolysis of dichlobenil, bromoxynil, chloroxynil, and ioxynil. The abbreviations are as follows: BAM: 2,6-dichlorobenzamide; 2,6-DCBA: 2,6-dichlorobenzoic acid; BrAM: 3,5-dibromo-4-hydroxy-benzamide; BrAC: 3,5-dibromo-4-hydroxybenzoic acid; IAM: 3,5-diiodo-4-hydroxybenzamide; and IAC: 3,5-diiodo-4-hydroxybenzoic acid. Degradation pathway utilizing enzyme nitrilase has not been previously observed for dichlobenil [3].

Every year about six thousand of tons of organic herbicides are deliberately introduced into the environment in order to prevent loss of cultural crops. This paper aims to evaluate the degree of hazard of the benzonitrile herbicides. Two substances of this herbicidal group, bromoxynil and ioxynil, are currently approved for commercial use in the European Union including the Czech Republic. Dichlobenil was prohibited as an herbicide by the European Union in the year 2008 [1]. Besides the high risks of dichlobenil used in granular form for aquatic environment and birds, one of the main reasons for the ban of the herbicide was the presence of high amounts of its metabolic product 2,6-dichlorobenzamide (BAM) generated by microorganisms (Figure 1) [2]. Most recently, these metabolites, in particular amides and acids of original substances, have been studied intensively because of their possible toxic effects [3].

Bromoxynil, chloroxynil, dichlobenil, and ioxynil are structurally similar members of the benzonitrile herbicides group. Except chloroxynil, they have been widely used in agriculture and households to control growth of weeds and their residues persist in the environment. The mechanism of toxic effects to target organisms was studied in detail in bromoxynil, dichlobenil, and ioxynil. Bromoxynil and ioxynil belong to the so-called mitochondrial uncouplers, which are relatively simple but very effective toxicants. Uncouplers generally act as protonophores which carry protons across the impermeable inner membrane of mitochondria by means of electrical and chemical gradients, which they are able to create [4]. In addition to the fact that both herbicides damage the system of oxidative phosphorylation and inhibit mitochondrial activity, they were also found to inhibit the activity of chloroplasts [5–8]. Dichlobenil is ranked among inhibitors of plant cell wall biosynthesis [9]. Toxicity of the three used benzonitrile herbicides, bromoxynil, dichlobenil, and ioxynil, was tested in many prokaryotic and eukaryotic systems. The results of these tests provide detailed information on acute and chronic toxicity, carcinogenicity, and mutagenicity [10, 11]. In contrast, there is very little information available for the toxicity and mutagenicity of the fourth herbicide, chloroxynil, due to the fact that this substance is not used because of its low herbicidal properties. Ioxynil was reported as strong inhibitor of triiodothyronine binding to transthyretin, one of the thyroid hormones binding proteins [12, 13]. Obviously, differences in chemical structures and properties of benzonitrile herbicides may have an impact on their degradation and spreading in the environment. Bromoxynil, chloroxynil, and ioxynil, unlike the relatively stable dichlobenil, are more susceptible to photolysis due to their hydroxyl group [3]. Dichlobenil and ioxynil also easily undergo hydrolysis in alkaline medium [3]. There are a number of studies dealing with photodegradation of benzonitrile herbicides under different conditions [14–16]; however, it can be said that abiotic degradation of these substances is less effective than microbial degradation [3]. That is why the biodegradation of benzonitrile herbicides by soil microorganisms has been studied intensively [17].

The main products of bromoxynil biodegradation in soil (Figure 1), detected in most biodegradation experiments, are 3,5-dibromo-4-hydroxybenzamide (BRAM) and 3,5-dibromo-4-hydroxybenzoic acid (BRAC) [18, 19]. Dichlobenil is degraded in soil [20] and subsurface sediments [21]. It was found that this herbicide is hydrolysed most often by the enzymes nitrilase, amidase, and nitrilhydratase, generating 2,6-dichlorobenzamide (BAM) and 2,6-dichlorobenzoic acid (DCBA) [3, 22, 23]. Besides hydrolysis, another option of dichlobenil decomposition is reductive dechlorination. Given the widespread use of dichlobenil and the persistence of its amide (BAM) and its presence in groundwater, the distribution of both of these substances in the environment is monitored in many countries. Their concentrations are observed not only in water and soil, but also in the air and living organisms [24]. Ioxynil is well biodegradable in soil and detected metabolites (Figure 1) are similar to those formed in the case of bromoxynil [25]. Complete mineralization of the ioxynil to CO_2_ was also observed [26]. The ioxynil degradation to 3,5-diiodo-4-hydroxybenzoic acid (IOXAC) was observed in the strains* Rhodococcus *sp. NDB 1165,* Nocardia globerula* NHB-2, and* Rhodococcus rhodochrous* PA-34. Based on the information published by Veselà et al. [17], chloroxynil is hydrolyzed in the same manner as the structurally similar bromoxynil and ioxynil. In this experiment, the 3,5-dichloro-4-hydroxybenzoic acid (CHXAC) was identified as a decomposition product by* Rhodococcus *sp. NDB 1165.

Our study focuses on determination of the cytotoxicity of benzonitrile herbicides and their metabolites which could arise by their bacterial degradation. Two human cell lines derived from potential xenobiotic target organs in humans, that is, Hep G2 and HEK293T (cells reflecting liver and kidneys, resp.) [27], were chosen to assess their potential cytotoxic effects.

## 2. Materials and Methods

### 2.1. Chemicals

The substrates of analytical grade purity, benzonitrile, 3,5-dichloro-4-hydroxybenzonitrile (chloroxynil), 3,5-dibromo-4-hydroxybenzonitrile (bromoxynil), 3,5-diiodo-4-hydroxybenzonitrile (ioxynil), and 2,6-dichlorobenzonitrile (dichlobenil), and the standards of their biotransformation, benzamide, benzoic acid, 3,5-dichloro-4-hydroxybenzoic acid, 3,5-dibromo-4-hydroxybenzoic acid, 3,5-diiodo-4-hydroxybenzoic acid, 2,6-dichlorobenzamide, and 2,6-dichlorobenzoic acid, were purchased from standard commercial sources (Sigma Aldrich, Alfa Aesar). Authentic standards of 3,5-dichloro-4-hydroxybenzamide, 3,5-dibromo-4-hydroxybenzamide, and 3,5-diiodo-4-hydroxybenzamide were purchased from Shanghai Fangkai Chemical (China). All substances were dissolved in methanol 99.8% in concentration of 1 g/L. At this concentration all the tested compounds are soluble.

### 2.2. Cell Cultures

Hep G2-ATCC HB-8065 cells are derived from human hepatocellular carcinoma and HEK293T-ATCC CRL-11268 are epithelial cells derived from kidney of human fetus. HEK293T cells were cultivated in Dulbecco's Modified Eagle's Medium (DMEM Sigma Aldrich; with 4.5 g/L glucose and L-glutamine) and Hep G2 in RPMI 1640 (Sigma Aldrich) medium, both of them supplemented with 10% fetal bovine serum (FBS) and 1% of MEM (mix of vitamins, Gibco, GB). Cells were cultured at 37°C and 5% CO_2_. The generation time (the duration of one cycle of cell cycle) of Hep G2 cell line is 48 h and the generation time of HEK293T cells is 24 h [28, 29].

### 2.3. Monitoring of Cell Growth with the xCELLigence RTCA DP Instrument

Experiments were carried out using the xCELLigence RTCA DP Instrument (Roche Diagnostics GmbH, Mannheim, Germany) which was placed into an incubator (37°C and 5% CO_2_). Cell proliferation and cytotoxicity experiments were performed using modified 16-well plates (E-plate, Roche Diagnostics GmbH, Mannheim, Germany). Microelectrodes are attached at the bottom of the wells for impedance-based detection of attachment, spreading, and proliferation of the cells. Initially, 100 *μ*L of cell-free growth medium with 10% fetal bovine serum (FBS) and 1% of MEM (mix of vitamins, Gibco, GB) was added to the wells for calibration.

Cells were harvested from exponential phase cultures by a standardized detachment procedure using 0.25% Trypsin-EDTA and the cell number was counted automatically using Roche's CASY Cell Counter and Analyzer. 100 *μ*L of the Hep G2 cell suspension at concentration of 5 × 10^5^ cells/mL and 10^6^ HEK293T cells/mL was seeded into the wells for cytotoxicity experiments.

Twenty-four hours after cell seeding, tested substances dissolved in methanol were added (to final concentrations of 10, 25, 50, and 100 mg/L). PBS and methanol alone were added to control wells. Each concentration was tested in duplicate within the same experiment. CI (cell index) was monitored every 60 min during the experiment for 72 hours. A dimensionless parameter corresponding to the relative change in measured electrical impedance represents cell status (number of attached cells). These results were transferred into growth curves (dependence of the impedance expressed by the “cell index” value on time) of cells monitored in the presence of the tested compounds. The inhibitory factor *I* (%), reflecting growth inhibition activity of tested substances, was determined as well.

Data were analysed using the statistical and graphical functions of RTCA Software v1.2 (ACEA Biosciences Inc., USA). The statistical significance of results was tested by Welsh's *t*-test on exported raw data in R-project (http://www.R-project.org/). For all statistical tests, the significance level was established at *P* < 0.05.

### 2.4. MTT Test (The Cell Proliferation Kit I, Roche)

Hep G2 and HEK293T cells were seeded at densities of 5 × 10^5^ cells/mL and 10^6^ cells/mL, respectively, into wells of 96-well plates and cultivated for 24 h before pesticide exposure. The cells were then treated with 50 *μ*L of different concentrations (10, 25, 50, and 100 mg/L) of tested substances. Due to their different generation time, the HEK293T cells were exposed to 24 h and Hep G2 cells for 48 h. At the end of the exposure time, 10 *μ*L of MTT solution was added to each well and incubated for 30 min at 15–20°C. The plates were then placed into the cell culture incubator (5% CO_2_ and 37°C) for additional 4 h. The MTT solution was removed and 100 *μ*L of DMSO was added to each well to dissolve the blue formazan crystals. The optical density at 600 nm was measured (using UV-Vis DU 700 spectrophotometer, Beckman Coulter, USA). All assays were performed in triplicate.

### 2.5. Lactate Dehydrogenase (LDH) Test

The test was performed using the Cytotoxicity Detection Kit^PLUS^ (Roche). Both cells lines were seeded at a density of 10^5^ cells/mL in wells of 96-well plates and cultivated for 24 h. The cells were then treated with 50 *μ*L of different concentrations (final concentrations 10, 25, 50, and 100 mg/L) of tested substances. To determine the LDH concentration, 24 h after treatment for HEK-293T cells and 48 h after treatment for Hep G2 cells, 100 *μ*L of Cytotoxicity Detection Kit^PLUS^ reaction mixture was added to each well and incubated for up to 30 min at 15–20°C. The absorbance at 490 nm was measured using a spectrophotometer (UV-Vis DU 700, Beckman Coulter). The reference wavelength was 690 nm. All assays were performed in triplicate.

## 3. Results

### 3.1. Proliferation of Exposed Cell Lines

Selected cell lines (Hep G2 and HEK293T) were exposed to all benzonitrile herbicides (bromoxynil, chloroxynil, dichlobenil, and ioxynil) and to products of their microbial degradation (acids and amides, Figure 1).

**Figure 2:**
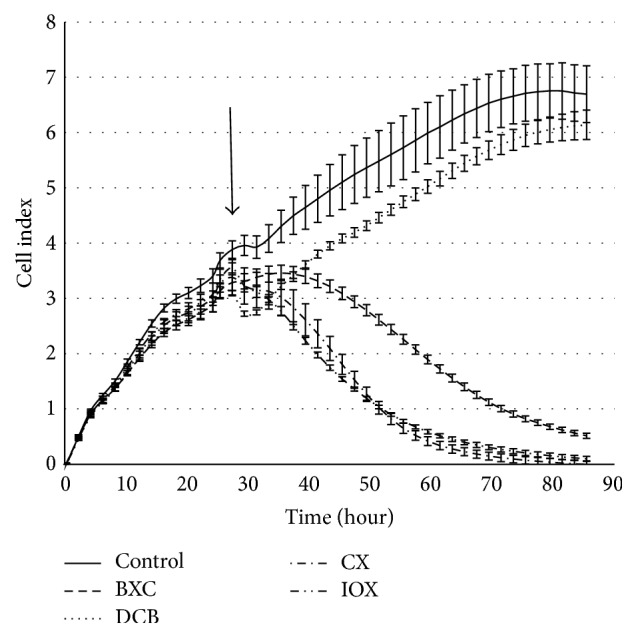
The growth curves of Hep G2 cells in the presence of benzonitrile herbicides (100 mg/L). The arrow indicates the time of the application of tested compounds. Error bars are standard deviation of 3 parallels.

The comparison of the growth curves of Hep G2 cells exposed to the tested substances (Figure 2) shows a significant (*P* < 0.05) cytotoxic effect of three herbicides: bromoxynil, chloroxynil, and ioxynil.

At the highest tested concentration, the cytotoxic effect of ioxynil, bromoxynil, and chloroxynil appeared 18 h after application (see Figure 2). Since then, the viability of cells treated by chloroxynil, ioxynil, and bromoxynil continued to gradually decrease over a period of 48 h when all the cells detached from the support. Interestingly, dichlobenil did not show any cytotoxicity measured using impedance-based detection (xCELLigence RTCA DP Instrument). For better clarity of cytotoxic effects of original compounds and their metabolites, the data shown as viability curves are presented also as inhibitory factor *I* (%), which reflects growth inhibition activity of the tested substances. To evaluate potential risk of metabolites derived from the parent herbicides, the inhibitory factor values were determined 24 h and 48 h after treatment for both the tested herbicides and their metabolites (listed in Table 1). The viability curves for metabolites of the herbicides were measured as well (data not shown) and the results were converted to inhibitory factor. As shown in Table 1, both acids and amides derived from the pesticides appeared to be less toxic to the Hep G2 cells than the original compounds (bromoxynil, chloroxynil, and ioxynil) with both the *I*_24_ and *I*_48_ values ranging between 90 and 100% at the 100 mg/L concentration (Table 1). The dichlobenil showed only limited toxicity to the Hep G2 cells at higher concentrations (50 and 100 mg/L) and no growth inhibition was observed at the concentration 25 mg/L.

All the metabolites originating from parent herbicides (dichlobenil, bromoxynil, and ioxynil) showed only slight inhibition (less than 20%) at the concentration 100 mg/L. The only exception was 3,5-dichloro-4-hydroxybenzoic acid (CHXAC), the metabolic product of chloroxynil, to which Hep G2 cells at the concentration 100 mg/L exhibited the inhibitory index value 56% after 48 h exposure (Table 1).

**Figure 3:**
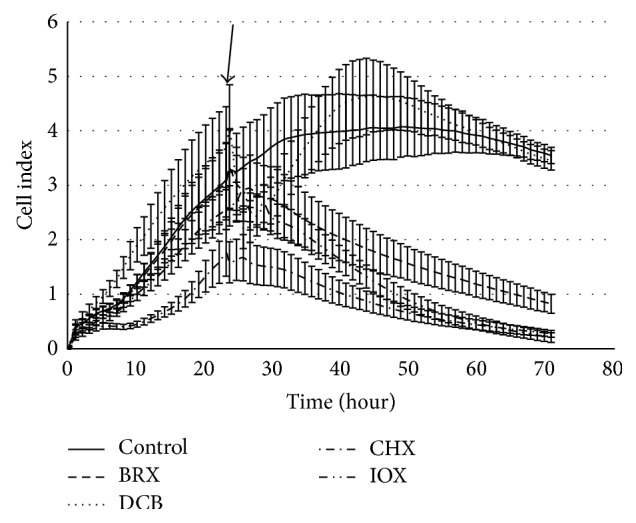
The growth curves of HEK293T cells in the presence of benzonitrile herbicides (100 mg/L). The arrow indicates the time of the application of tested compounds. Error bars are standard deviation of 3 parallels.

The cytotoxic effect of all four benzonitrile herbicides and their metabolites was tested also on a kidney cell line, HEK293T cells. The viability curves shown in Figure 3 are similar to those measured on Hep G2. As expected, the cell proliferation decrease caused by CHX, BRX, and IOX was concentration dependent as shown for concentrations 25 mg/L, 50 mg/L, and 100 mg/L (Table 2).

None of the metabolites induced any toxicity response. In general, the values of inhibitory factors of BRX, CHX, and IOX are lower for HEK293T cell in comparison to values measured on Hep G2 cells. At the concentration 100 mg/mL (the highest used), the inhibition effect of both IOX and BRX on the HEK293T cells was lower by 33% in comparison to Hep G2 cells. Similar difference, although slightly lower, was observed for CHX which showed by 20% lower inhibition of the HEK 293T viability in comparison to the Hep G2 cells. The inhibitory factor value of CHXAM, which is a metabolite of CHX, is for the HEK 293T cells similar with that obtained for Hep G2 cells. At the highest used concentration (100 mg/mL) the inhibitory factor value of CHXAM was 12%. Both DCB and metabolites derived from all the tested herbicides showed no toxicity response.

### 3.2. MTT Assay

The inhibition effect of tested substances was determined also by MTT test monitoring the cell viability. The data obtained by the MTT assay for all original herbicides and for all their tested metabolites on Hep G2 and HEK293T cell lines are summarized in Table 3.

These herbicides (at the concentration of 100 mg/L), except the dichlobenil, showed the inhibition of viability of both tested cell lines. Their inhibitory coefficient values were lower on HEK293T cells (varied between 39% and 61%) in comparison with the effect on Hep G2 cells with *I*_48_ higher than 91% (Table 3). The same trend in inhibition differences was also observed in proliferation test measured by xCELLigence RTCA DP Instrument. Additionally, the nontoxic effect of dichlobenil was also confirmed, which is in good agreement with the data shown in Tables 1 and 2.

In comparison to the parent herbicides, all their tested metabolites showed only limited viability inhibition at concentration 100 mg/L. The inhibition coefficient values of the metabolites were similar for both tested cell lines and varied in the range of 13 to 22% for Hep G2 and 7 to 25% for HEK293T. Thus, MTT test, which monitors the cell viability, also proved that metabolic products are less toxic compared to their original compounds.

### 3.3. Detection of Cytotoxicity by Lactate Dehydrogenase Test

The cytotoxicity of the tested substances on the Hep G2 and HEK293T cells was determined also by using lactate dehydrogenase (LDH) Cytotoxicity Detection Kit^PLUS^, Roche. The LDH assay was performed for all the tested substances in three parallels at concentrations of 100, 50, and 25 mg/L. The HEK293T and Hep G2 cells were treated by the tested substances for 24 and 48 hours, respectively. The LDH assay determines the mortality expressed as the percentage of the dead cells by addressing the damage of the cell membrane integrity monitored as leakage of intracellular lactate dehydrogenase. As shown in Table 4, the LDH assay indicated that the three herbicides, bromoxynil, chloroxynil, and ioxynil, at the concentration of 100 mg/L have a significant cytotoxic effect on Hep G2 cell line.

The remaining substances, dichlobenil and microbial metabolites of all the four herbicides, did not show any cytotoxicity detectable by this test. This is in general agreement with the results described above for Hep G2 cells tested by xCELLigence RTCA DP Instrument and MTT assay, where high cytotoxic effect was observed especially for BRX, CHX, and IOX (see Figures 2 and 3 and Tables 1, 2, and 3). By using the LDH assay, we did not detect any effect of the tested substances on HEK293T cells. These differences may indicate different sensitivity of the cell lines used in this study as explained in the discussion. It also underlines a necessity to determine cytotoxic effect by using rationally selected cell lines and by various methods targeting different cellular features, as the cell viability, proliferation, and membrane integrity, used in our case.

## 4. Discussion

An intensive use of benzonitrile herbicides led to their widespread occurrence in the environment [24, 30, 31]. However, the toxicity data of these compounds and their metabolic products for humans is still limited. In our study, the cytotoxic effects of dichlobenil, bromoxynil, chloroxynil, and ioxynil and their metabolites were examined by three different methods (xCELLigence RTCA DP Instrument, MTT assay, and LDH assay) on two cell lines. As the liver and kidneys are the main detoxification organs, HEK293T (embryonal kidney cells) and Hep G2 (hepatocarcinoma cell line) cell line were selected as models for the toxicity evaluation experiments. Moreover, Hep G2, human liver carcinoma cell line, is a suitable model for toxicology studies, since their enzymatic equipment is very similar to hepatocytes [32] and HEK293T, human embryonic kidney cell line, is widely used as* in vitro* system for cytotoxicity testing [27, 33, 34]. It was shown previously that the Hep G2 cells produce a large set of enzymes involved in metabolism of xenobiotics. The presence of this enzymatic battery, which includes high levels of cytochromes P450, CYP1A2, CYP3A4, and glutathione S-transferase could be the reason for the higher sensitivity of the Hep G2 cell line to the tested herbicides [35, 36]. Also, we hypothesize that the tested substances could be transformed by this enzymatic machinery into products affecting expression of genes involved in stress response or other metabolic mechanisms leading to necrosis. This would explain why higher level of LDH was observed in cultivation media of the Hep G2 cells treated with bromoxynil, chloroxynil, and ioxynil at the concentration of 100 mg/L compared to the HEK293T cells. Interestingly, the level of cytochrome P450 is weaker in Hep G2 cells than in primary hepatocytes, which according to Westerink and Schoonen [37] could lead to some underestimation of real toxicity of tested substances. However, the cell line models are widely accepted for toxicity testing and similar experiments on primary hepatocytes would exceed our limits.

In both tested cell lines, significant cytotoxic effects were observed for three herbicides: bromoxynil, chloroxynil, and ioxynil at all tested concentrations (100 mg/L, 50 mg/L, and 25 mg/L). In contrast, dichlobenil was almost nontoxic even at the highest tested concentration, that is, 100 mg/L. These results are consistent with other studies [3, 38, 39] where dichlobenil exhibited less acute toxicity to mammals (LD_50_ 1014–4460 mg/kg bw) than bromoxynil (LD_50_ 81–260 mg/kg bw) and ioxynil (LD_50_ 110–230 mg/kg bw). A similar acute toxicity impact of these herbicides was also shown for birds, for example, bobwhite quail, when dichlobenil exhibited lower toxicity (LD_50_ 698 mg/kg bw) than bromoxynil (217 mg/kg bw). The different mechanism of action of these compounds may be the reason why the cells responded differently. While bromoxynil and ioxynil inhibit mitochondrial activity and oxidative phosphorylation [4], dichlobenil inhibits the biosynthesis of cellulose [40].

Our reported cytotoxic concentrations are severalfold higher than European Union administrative threshold limits for single pesticide in ground water and drinking water which is 0.1 *μ*g/L and the total sum of pesticides and their metabolites must not exceed 0.5 *μ*g/L [41]. However, in areas with herbicide large-scale soil applications, the concentrations of these herbicides or their metabolites were detected as considerably higher, reaching up to concentration 560 *μ*g/L. But this concentration is exceptional and even levels above 10 *μ*g/L were observed rarely [30]. The concentration in sediments and wetlands are in low range due to the dissipation behavior in soil [21, 24, 42].

Bromoxynil and ioxynil are currently approved as herbicidal products for commercial use in the European Union. For example, bromoxynil is allowed for use on fields with crops serving as a green fodder for livestock. Therefore, European Food Safety Authority (EFSA) reviewed the Maximum Residue Levels (MRLs) for the bromoxynil in order to assess the bromoxynil residues in plants, processed commodities, rotational crops, and livestock and concluded that the use of bromoxynil on crops did not indicate risk to consumers [43]. However, it is known that pesticides contaminate river and lake waters due to spills from agricultural use and therefore bioconcentration in food chain could pose the risks [44]. The bioconcentration factors (BCFs, the ratio of the chemical concentration in an organism to the concentration in water) for dichlobenil ranged from 32, 63, and 110 for fillet, whole fish and viscera, respectively, with high rate of depuration [45]. The BCFs for ioxynil in fish were reported in range 6 to 29 [46] and bromoxynil has low log Pow (partition coefficient); therefore their BCFs are expected to be low [47]. Although reported BCFs of the studied pesticides are low, cumulative effect could emerge under a chronic exposition to these pesticides. The other case can be an accidental administration. An example of the latter is described in a paper published by Berling et al. [48] who reported fatal case due to ingestion of MCPA/bromoxynil coformulation herbicide. Two hours after the ingestion, the concentration of bromoxynil was reported as 137 mg/L in blood, which is comparable with concentration used in our study. Sadly, the men died 18 h after exposure.

The purpose of this study was also to evaluate the potential toxicity risks of metabolic products of original herbicides (Figure 1) that may be formed by microorganisms in the contaminated environment [3]. There have been reported several groups of contaminants whose degradation products exhibit higher toxicity than the original compound. For example, this is the case of widely used brominated flame retardant BDE 209 which is degraded by bacteria to more toxic lower brominated congeners [49, 50].

Microbial metabolites of bromoxynil, chloroxynil, and ioxynil exhibited lower cytotoxicity compared to parental compounds (Tables 1 and 2). The exception was 3,5-dichloro-4-hydroxybenzoic acid (CHXAC), which inhibited proliferation of the Hep G2 cells at the concentration of 100 mg/L (measured by the xCELLigence RTCA DP Instrument). However, the other tests (MTT and LDH assays) did not show the toxic effects. It should be emphasized that our knowledge concerning toxicity of metabolic product of bromoxynil, chloroxynil, and ioxynil is limited due to the few experiments conducted in this area. Veselá et al. [17] described acute toxicity of these metabolic products using bacterium* Vibrio fischeri *bioluminescence test and measuring inhibition of* Lactuca sativa *germination. Among tested metabolites, DCBA and BAM showed the highest toxic effects, as indicated by the bioluminescence and EC_50_ values that were 1773 and 505 *μ*M, respectively. The highest inhibition of germination was noticed for dichlobenil. Some studies are devoted to the dichlobenil metabolic product 2,6-dichlorobenzamide (BAM), which is on interest due to its higher persistency and mobility in the environment compared to dichlobenil [51]. BAM inhibits the production of chlorophyll, and therefore the chlorosis was observed as a side effect in plants and algae with EC_50_ for* Chlorella pyrenoidosa* 100 mg/L [52, 53].

## 5. Conclusion

It is known that some original contaminants can be less toxic than the byproducts originating from their biodegradation/biotransformation. Therefore the present study is devoted to the cytotoxicity of both parent herbicides and their known metabolites which could arise in soil or water environment by microbial degradation. Our results demonstrate that all these metabolic products are less cytotoxic than their parent compounds. More importantly, these data fill the gap in the knowledge of toxicity of the metabolic products of currently used herbicides, bromoxynil and ioxynil, and thus confirm safety of their applications. In conclusion, the bromoxynil, chloroxynil, and ioxynil showed only moderate cytotoxic effects at concentrations several times higher than their limits allowed in the drinking water. Their degradation products were nontoxic. However, this does not exclude their potential risk connected with chronic exposure to some of these compounds.

## Figures and Tables

**Table 1:** The inhibition coefficients *I*_24_ (%) for 24 h exposure and *I*_48_ (%) for 48 h exposure of benzonitrile pesticides and their microbial metabolic products on Hep G2 cells for concentrations 25 mg/L, 50 mg/L, and 100 mg/L.

Compounds	*I*_24_ (%) 25 mg/L	*I*_48_ (%) 25 mg/L	*I*_24_ (%) 50 mg/L	*I*_48_ (%) 50 mg/L	*I*_24_ (%) 100 mg/L	*I*_48_ (%) 100 mg/L
BRX	20 ± 1	45 ± 3	52 ± 1	77 ± 2	92 ± 4	95 ± 3
BRAC	8 ± 0,4	11 ± 1	10 ± 1	10 ± 1	15 ± 1	20 ± 2
BRAM	ND	ND	ND	9 ± 0,5	ND	12 ± 2
CHX	50 ± 3	72 ± 2	70 ± 4	90 ± 2	90 ± 2	99 ± 1
CHXAC	10 ± 1	17 ± 2	25 ± 3	35 ± 2	50 ± 2	56 ± 3
CHXAM	ND	9 ± 1	ND	4 ± 0,1	10 ± 1	13 ± 1
DCB	ND	ND	ND^*∗*^	14 ± 1^*∗*^	ND^*∗*^	15 ± 2^*∗*^
DCBA	ND	ND	ND	ND	20 ± 2	12 ± 1
BAM	ND	3 ± 0,2	ND	4 ± 0,2	10 ± 1	13 ± 3
IOX	50 ± 3	58 ± 3	70 ± 4	88 ± 5	100 ± 5	100 ± 4
IOXAC	ND	3 ± 0,2	ND	10 ± 2	ND	19 ± 2
IOXAM	ND	ND	ND	ND	ND	16 ± 1

BRX: 3,5-dibromo-4-hydroxybenzonitrile, BRAC: 3,5-dibromo-4-hydroxybenzoic acid, BRAM: 3,5-dibromo-4-hydroxybenzamide, DCB: 2,6-dichlorobenzonitrile, DCBA: 2,6-dichlorobenzoic acid, BAM: 2,6-dichlorobenzamide, CHX: 3,5-dichloro-4-hydroxybenzonitrile, CHXAC: 3,5-dichloro-4-hydroxybenzoic acid, CHXAM: 3,5-dichloro-4-hydroxybenzamide, IOX: 3,5-diiodo-4-hydroxybenzonitrile, IOXAC: 3,5-diiodo-4-hydroxybenzoic acid, and IOXAM: 3,5-diiodo-4-hydroxybenzamide; ND: below detection limit; *∗*: the solubility of DCB in water is around 20 mg/L; therefore the inhibitions at concentrations 50 and 100 mg/L are only indicative; ±: standard deviation of 3 parallels.

**Table 2:** The inhibition coefficient *I*_24_ (%) for 24 h exposure and *I*_48_ (%) for 48 h exposure for reflecting effect of benzonitrile pesticides on HEK293T cells for concentrations 25 mg/L, 50 mg/L, and 100 mg/L.

Compounds	*I*_24_ (%) 25 mg/L	*I*_48_ (%) 25 mg/L	*I*_24_ (%) 50 mg/L	*I*_48_ (%) 50 mg/L	*I*_24_ (%) 100 mg/L	*I*_48_ (%) 100 mg/L
BRX	34 ± 1	38 ± 2	52 ± 2	59 ± 2	52 ± 2	62 ± 2
CHX	68 ± 2	70 ± 1	70 ± 3	83 ± 3	70 ± 1	82 ± 4
IOX	48 ± 1	54 ± 2	59 ± 2	62 ± 3	59 ± 2	75 ± 3
DCB	ND	ND	ND^*∗*^	ND^*∗*^	ND^*∗*^	ND^*∗*^

BRX: 3,5-dibromo-4-hydroxybenzonitrile, CHX: 3,5-dichloro-4-hydroxybenzonitrile, IOX: 3,5-diiodo-4-hydroxybenzonitrile, and DCB: 2,6-dichlorobenzonitrile; ND: cytotoxicity not observed; *∗*: the solubility of DCB in water is around 20 mg/L; therefore the inhibitions at concentrations 50 and 100 mg/L are only indicative; ±: standard deviation of 3 parallels.

**Table 3:** The inhibition coefficient *I* (%) determined by the MTT test for Hep G2 and HEK293T cells for 25, 50, and 100 mg/L. The HEK293T cells were exposed for 24 h and Hep G2 cells for 48 h.

	*c* (mg/L)	Hep G2	HEK293T
		*I*_48_ (%)	*I*_24_(%)
BRX	100	91 ± 1	53 ± 2
	50	71 ± 1	25 ± 1
	25	28 ± 1	13 ± 3

BRAC	100	13 ± 3	7 ± 1
	50	15 ± 5	7 ± 1
	25	15 ± 7	10 ± 5

BRAM	100	17 ± 6	14 ± 1
	50	16 ± 3	12 ± 2
	25	8 ± 2	11 ± 4

DCB	100	9 ± 6^*∗*^	4 ± 4^*∗*^
	50	16 ± 8^*∗*^	9 ± 5^*∗*^
	25	21 ± 6	13 ± 7

DCBA	100	19 ± 4	10 ± 5
	50	21 ± 7	13 ± 5
	25	22 ± 3	9 ± 1

BAM	100	22 ± 5	12 ± 2
	50	20 ± 1	16 ± 2
	25	14 ± 4	14 ± 6

CHX	100	91 ± 1	39 ± 2
	50	74 ± 1	22 ± 4
	25	21 ± 3	19 ± 7

CHXAC	100	16 ± 6	18 ± 6
	50	17 ± 7	18 ± 7
	25	15 ± 4	19 ± 7

CHXAM	100	17 ± 7	22 ± 5
	50	19 ± 3	24 ± 4
	25	13 ± 2	16 ± 4

IOX	100	98 ± 1	61 ± 2
	50	87 ± 1	41 ± 6
	25	82 ± 3	22 ± 4

IOXAC	100	13 ± 7	14 ± 2
	50	13 ± 3	15 ± 2
	25	15 ± 6	18 ± 2

IOXAM	100	21 ± 4	25 ± 4
	50	19 ± 3	19 ± 6
	25	11 ± 2	18 ± 4

BRX: 3,5-dibromo-4-hydroxybenzonitrile, BRAC: 3,5-dibromo-4-hydroxybenzoic acid, BRAM: 3,5-dibromo-4-hydroxybenzamide, DCB: 2,6-dichlorobenzonitrile, DCBA: 2,6-dichlorobenzoic acid, BAM: 2,6-dichlorobenzamide, CHX: 3,5-dichloro-4-hydroxybenzonitrile, CHXAC: 3,5-dichloro-4-hydroxybenzoic acid, CHXAM: 3,5-dichloro-4-hydroxybenzamide, IOX: 3,5-diiodo-4-hydroxybenzonitrile, IOXAC: 3,5-diiodo-4-hydroxybenzoic acid, and IOXAM: 3,5-diiodo-4-hydroxybenzamide; SD: standard deviation of 3 parallel measurements; *∗*: the solubility of DCB in water is around 20 mg/L; therefore the inhibitions at concentrations 50 and 100 mg/L are only indicative; ±: standard deviation of 3 parallels.

**Table 4:** The mortality values (%) of Hep G2 cells determined by the LDH for concentration 25, 50, and 100 mg/L after 48 h.

	100 mg/L	50 mg/L	25 mg/L
BRX	31 ± 9	5 ± 3	2 ± 1
CHX	48 ± 10	33 ± 9	5 ± 3
IOX	43 ± 12	22 ± 8	1 ± 2
DCB	ND	ND	ND

BRX: bromoxynil, CHX: chloroxynil, IOX: ioxynil, and DCB: 2,6-dichlorobenzonitrile; ND: cytotoxicity not observed; ±: standard deviation of 3 parallels.
